# Supplementary material for: Meta-analysis of the Parkinson’s disease gut microbiome suggests alterations linked to intestinal inflammation
Source: NPJ Parkinsons Dis. 2021 Mar 10;7:27. doi: 10.1038/s41531-021-00156-z (PMC7946946; doi:10.1038/s41531-021-00156-z)
Supplement: Supplementary file 1 — Supplementary Matrials [file 41531_2021_156_MOESM1_ESM.pdf]

Supplementary Materials for the manuscript:

Meta-analysis of the Parkinson's disease gut microbiome suggests alterations linked to intestinal inflammation

Stefano Romano, George M. Savva, Janis R. Bedarf, Ian G. Charles, Falk Hildebrand, Arjan Narbad

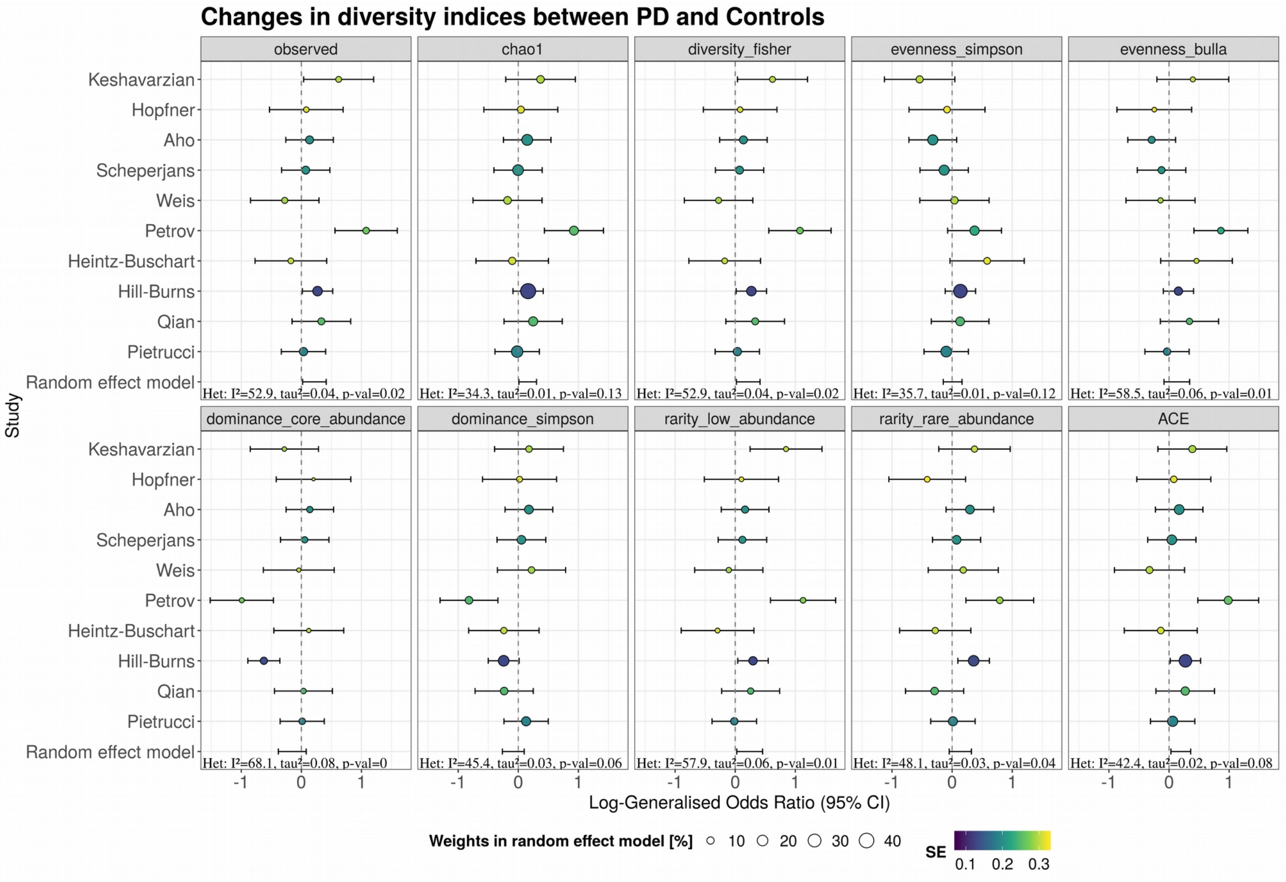

Supplementary Figure 1 | Forest plots reporting the fold changes of alpha-diversity indices across studies. For each dataset, fold changes were estimated using Agresti's generalized odd ratios. Estimators were then pooled using a random effect meta-analysis. The dashed line represents the line of no effect indicating the absence of differences between controls and PD patients. Indices higher in controls are shifted to the left, whereas indices higher in PD are shifted to the right. The size of the points indicates the weights given to each estimator in the meta-analysis approach. Heterogeneity (Het) of each dataset is reported in the bottom left of each panel.

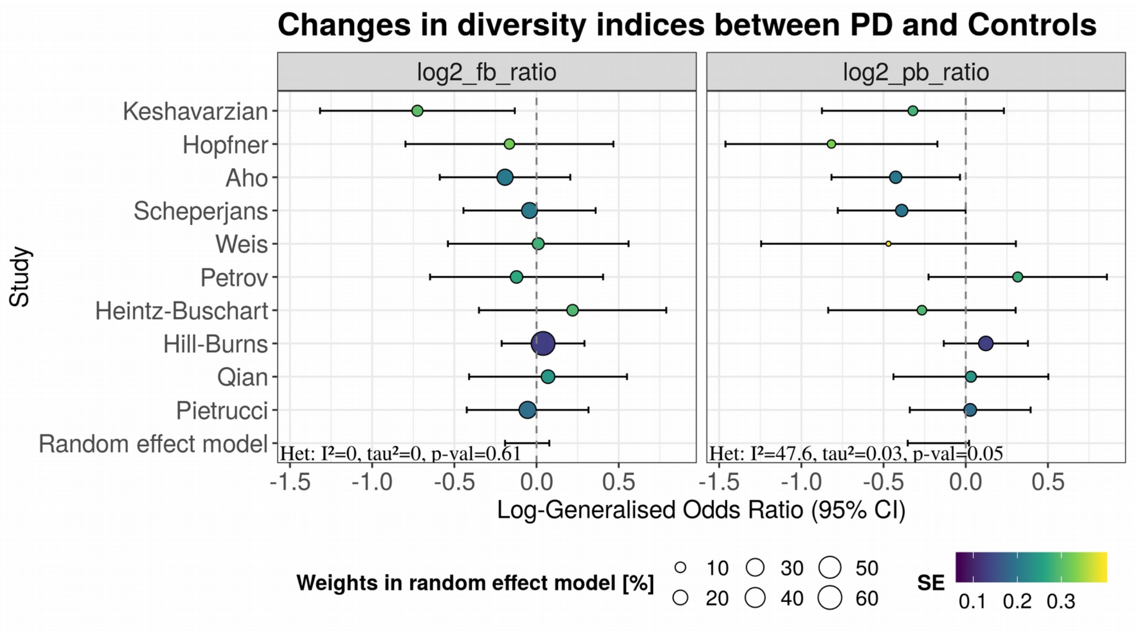

Supplementary Figure 2 | Forest plots reporting the fold changes of the Firmicutes to Bacteroidota (*Bacteroides* phylum) ratio (F/B ratio) and *Prevotella* to *Bacteroides* ratio (P/B ratio). For each dataset, fold changes were estimated using Agresti's generalized odd ratios. Estimators were then combined using a random effect meta-analysis. The dashed line represents the line of no effect indicating the absence of differences between controls and PD patients. Indices higher in controls are shifted to the left, whereas indices higher in PD are shifted to the right. The size of the points indicates the weights given to each estimator in the meta-analysis approach. Heterogeneity (Het) of each dataset is reported in the bottom left of each panel. No significant differences could be detected between controls and PD patients.

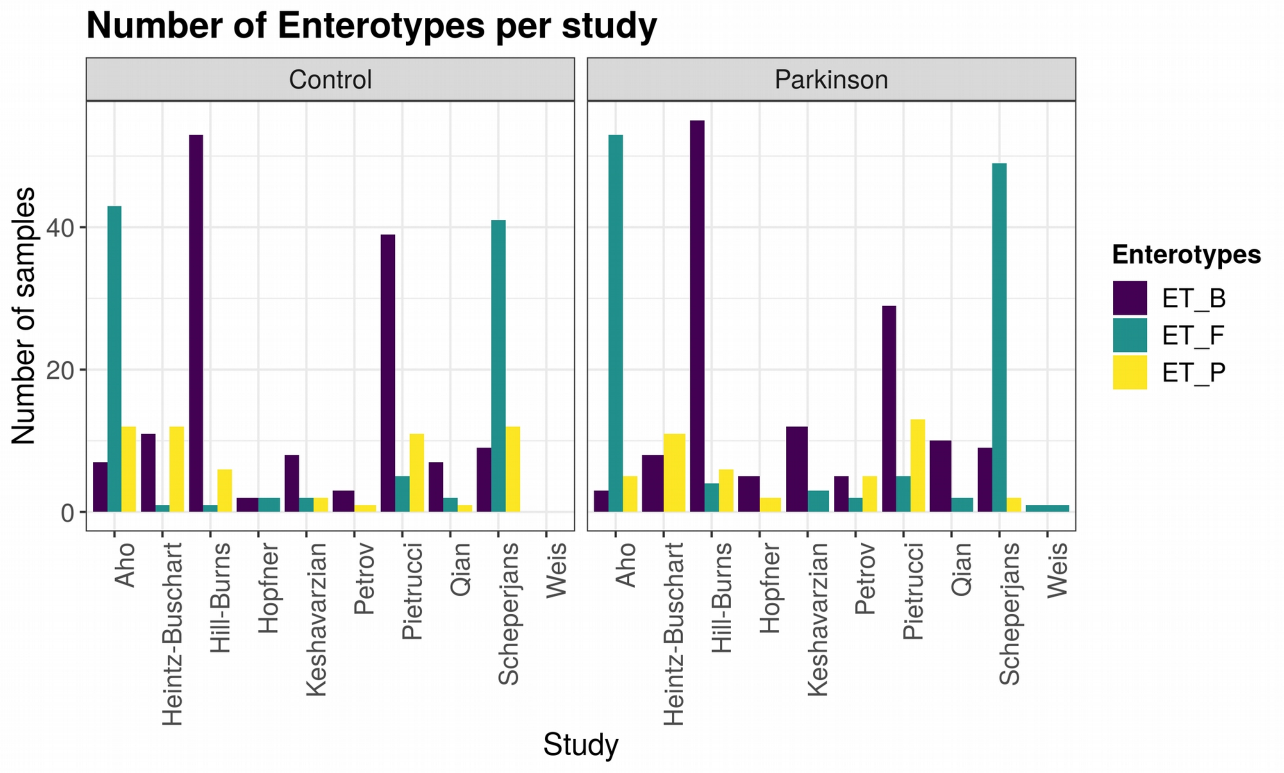

Supplementary Figure 3 | Distribution of samples across the three enterotypes. 592 samples could be assigned to known enterotypes. No clear differences were observed between PD patients and controls.

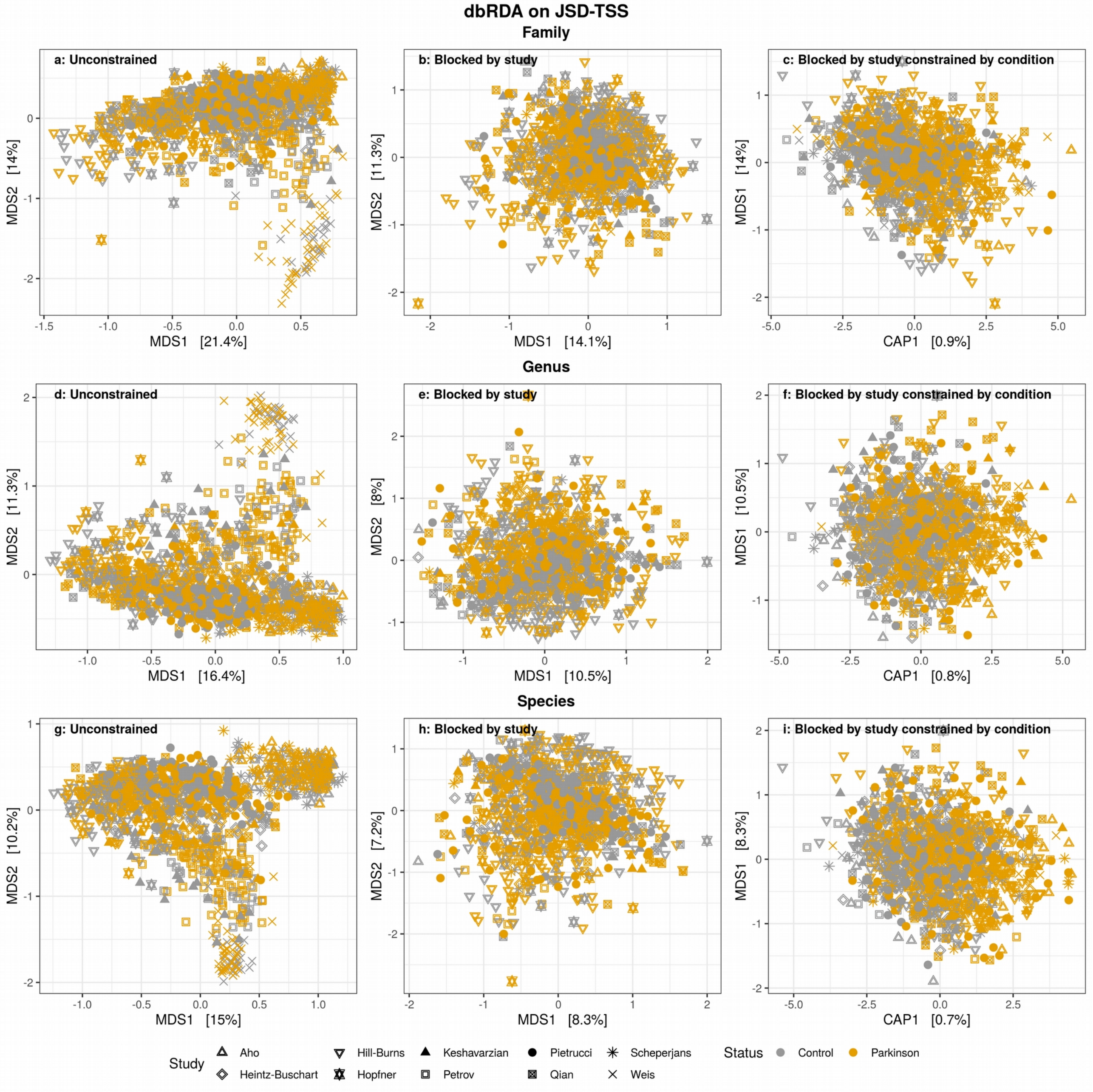

Supplementary Figure 4 | Bacterial community structures of the gut microbiome in PD patients and controls. Distance based redundancy analysis (dbRDA) was performed using the Jensen-Shannon divergence (JSD) on data normalized using total sum scaling (TSS). Clustering for family, genus and species are reported. We performed clusterings with no constrains and without accounting for the variation introduced by the studies (**a**, **d**, **g**); by removing (blocking) the variation explained by the studies (**b**, **e**, **h**); and by removing the variation introduced by the studies and constraining the data for disease status (**c**, **f**, **i**).

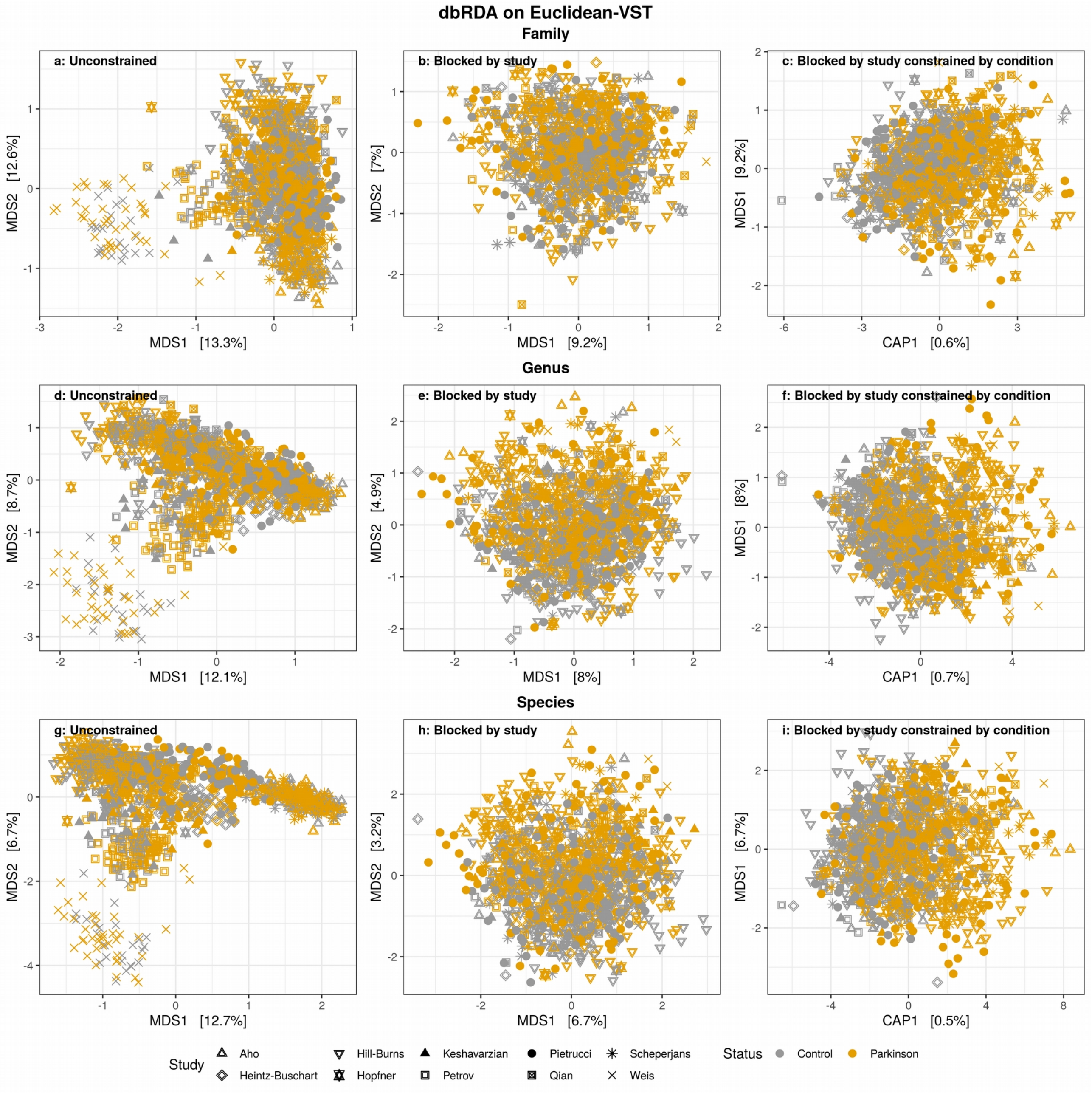

Supplementary Figure 5 | Bacterial community structures of the gut microbiome in PD patients and controls. Distance based redundancy analysis (dbRDA) was performed using Euclidean distances on data normalized using variance stabilizing transformation (VST). Clustering for family, genus and species are reported. We performed clusterings with no constrains and without accounting for the variation introduced by the studies (**a**, **d**, **g**); by removing (blocking) the variation explained by the studies (**b**, **e**, **h**); and by removing the variation introduced by the studies and constraining the data for disease status (**c**, **f**, **i**).

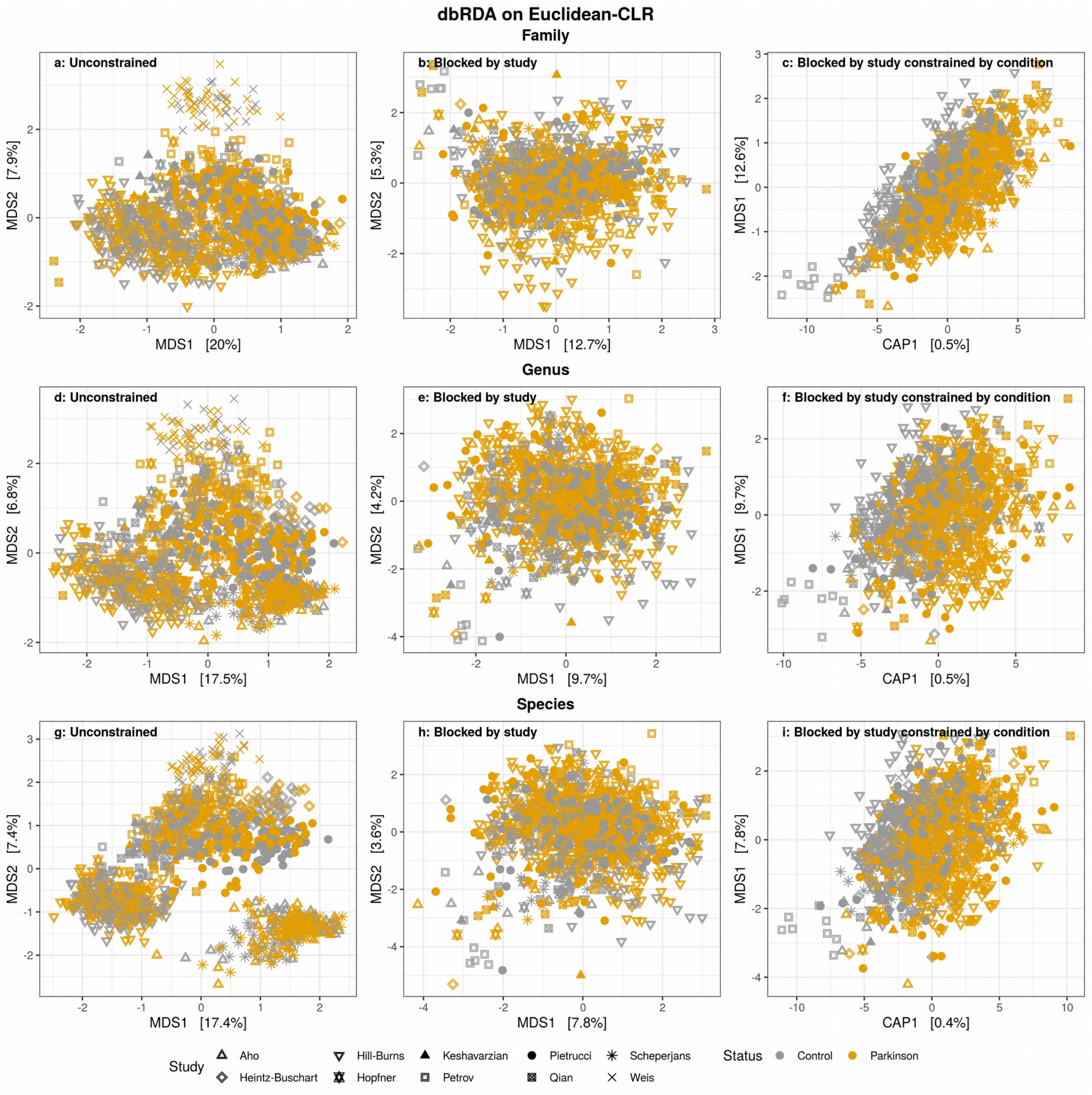

Supplementary Figure 6 | Bacterial community structures of the gut microbiome in PD patients and controls. Distance based redundancy analysis (dbRDA) was performed using Euclidean distances on data normalized using centered log-ratios (CLR). Clustering for family, genus and species are reported. We performed clusterings with no constrains and without accounting for the variation introduced by the studies (**a**, **d**, **g**); by removing (blocking) the variation explained by the studies (**b**, **e**, **h**); and by removing the variation introduced by the studies and constraining the data for disease status (**c**, **f**, **i**).

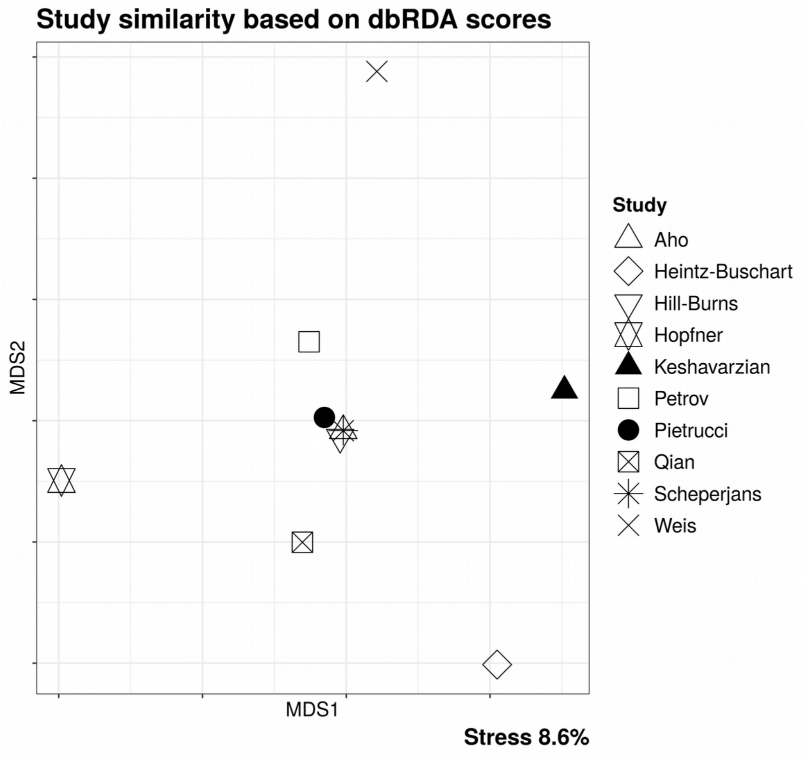

Supplementary Figure 7 | Similarity among studies. The sample coordinates obtained from the unconstrained and unconditioned dbRDA performed at the species level on data normalized using TSS were used to estimate distances among studies. The four most divergent studies used work-flows to process the samples that were markedly different from those used in the other six studies.

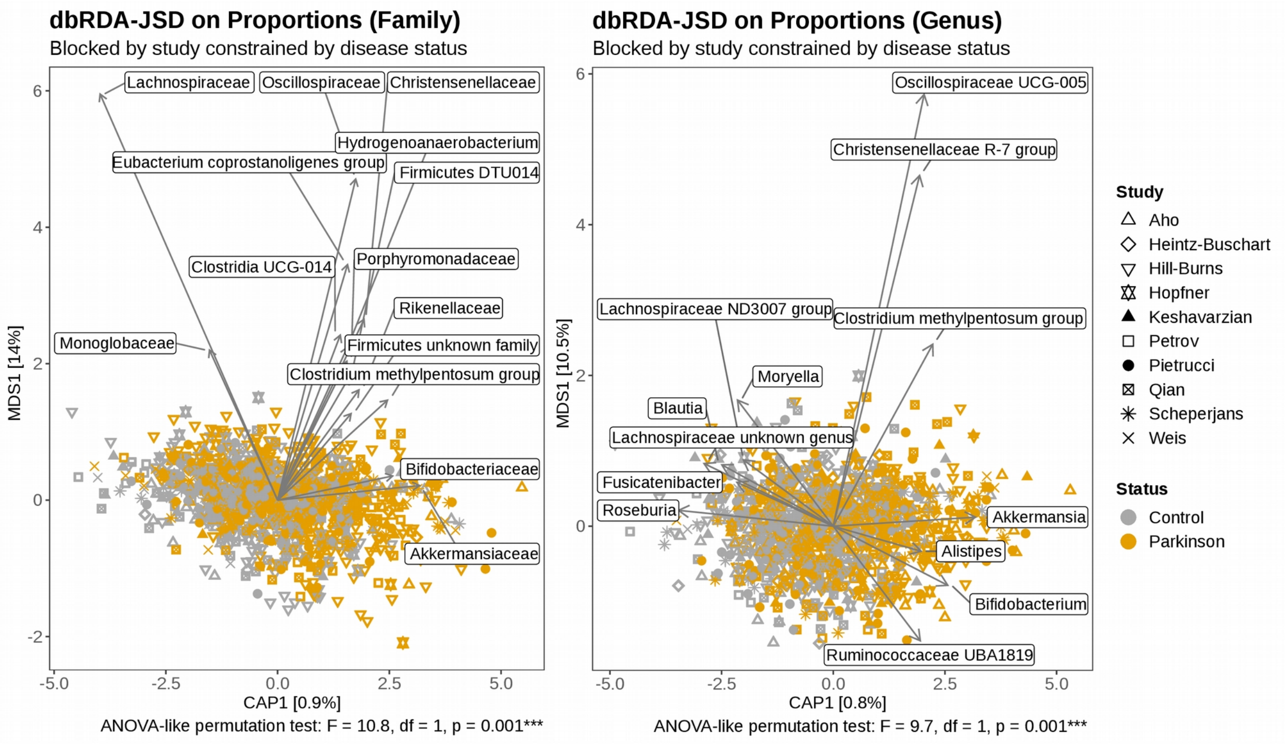

Supplementary Figure 8 | Most important families and genera influencing the divergence between PD and control samples. Distance-based redundancy analysis (dbRDA) was performed on Jensen-Shannon divergence (JSD) calculated on data normalized through total sum scaling (TSS). dbRDAs were conditioned by study and constrained by disease status. The limited proportion of data variability explained by the axis constrained for disease status (CAP1) indicates that environmental factors have a major influence in shaping the bacterial communities. However, the influence of the disease status on the community structure is statistically significant (ANOVA-like permutation test). Only taxa showing a significant association with the clustering of the samples and the strongest abundance variation between the conditions are reported.

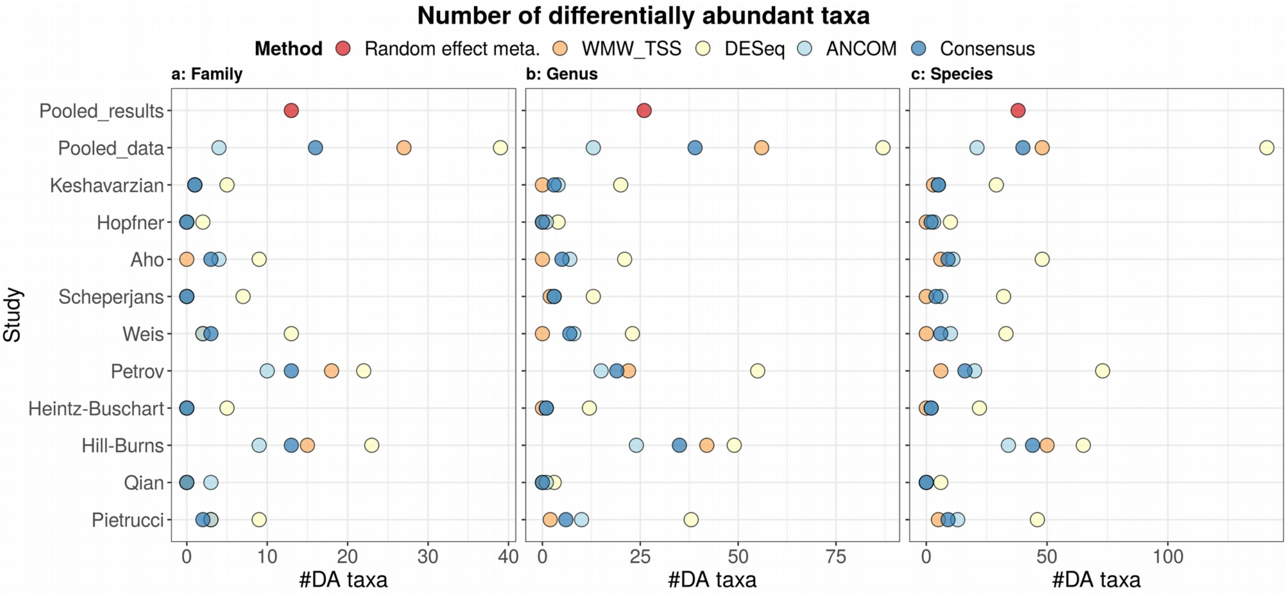

Supplementary Figure 9 | Number of taxa showing a significant difference in abundance between PD patients and controls. Data for family (**a**), genus (**b**), and species (**c**) are reported. Individual datasets were analyzed using three independent approaches: Wilcoxon-Mann-Whitney tests (MWM) on TSS normalized data; DESeq2; and ANCOM. The number of taxa showing differential abundance between conditions in at least two out of three methods is reported as “Consensus”. Significant differences were observed across studies and approaches. To obtain a global overview of the taxa differentially abundant in PD and controls we then used two distinct strategies: Pooled data and Pooled results approach. For the first, data were pooled and analyzed as reported above for the individual datasets. The “Consensus” of this analysis was then merged with the taxa of the Pooled results approach. In the latter, we normalized all datasets using CLR, tested differential abundance via linear models or Agresti’s generalized odd ratios, and pooled the results using random effect meta-analysis.

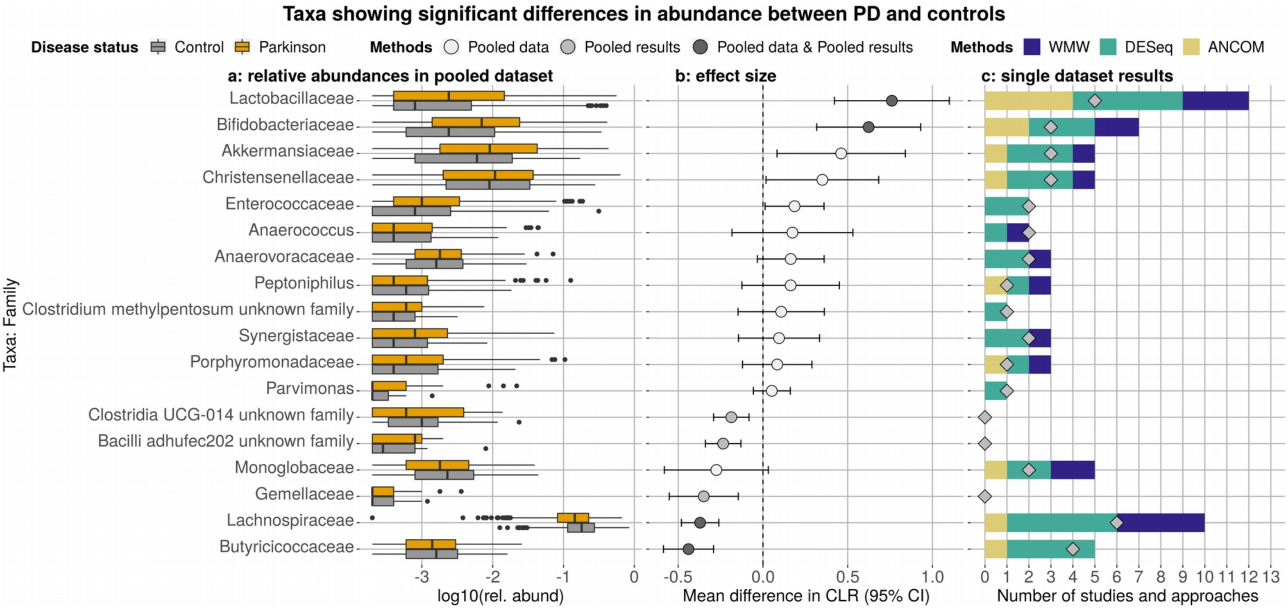

Supplementary Figure 10 | Families showing a significant difference in abundance between PD patients and controls. The relative abundances of the families retrieved from the rarefied pooled data are reported in panel **a**. Effect sizes were estimated via the mean difference in CLR (panel **b**) using a random effect meta-analysis approach (Pooled results approach). This was calculated for all taxa resulting differentially abundant in the Pooled results or Pooled data approaches. The color of the dots indicates which of the two above approaches detected the taxa differentially abundant. Taxa more abundant in controls have an effect size shifted to the left, whereas taxa more abundant in PD have an effect size shifted to the right. Panel **c** shows the number of times each family was detected differentially abundant between PD patients and controls across studies (diamonds) and approaches (bars). We used ten studies and three approaches, hence the maximum number of times a taxon can be detected differentially abundant is 30.

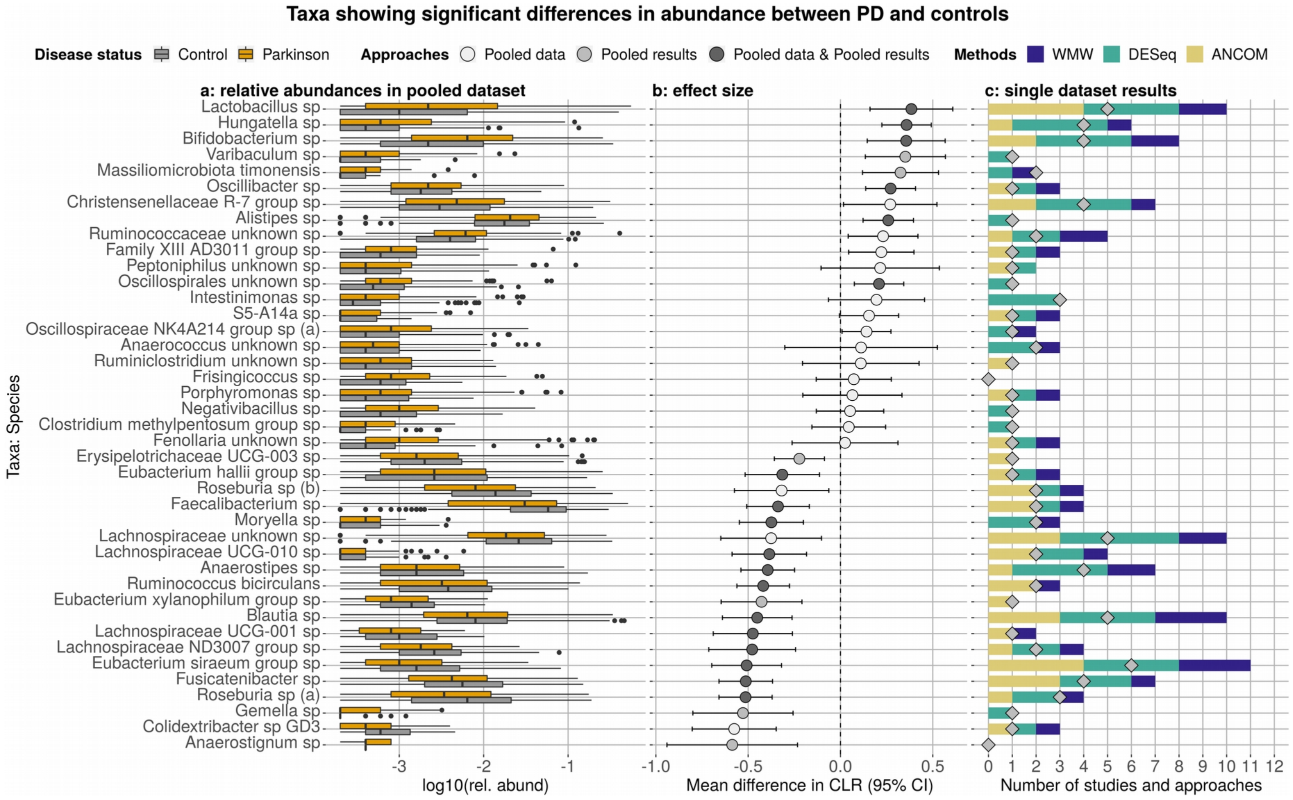

Supplementary Figure 11 | Species showing a significant difference in abundance between PD patients and controls. The relative abundances of the species retrieved from the pooled data are reported in panel **a**. Effect sizes were estimated via the mean difference in CLR (panel **b**) using a random effect meta-analysis approach (Pooled results approach). This was calculated for all taxa resulting differentially abundant in the Pooled results or Pooled data approaches. The color of the dots indicates which of the above two approaches detected the taxa differentially abundant. Taxa more abundant in controls have an effect size shifted to the left, whereas taxa more abundant in PD have an effect size shifted to the right. Panel **c** shows the number of times each species was detected differentially abundant between PD patients and controls in at least one method across studies (diamonds) and approaches (bars). We used ten studies and three approaches, hence the maximum number of times a taxon can be detected differentially abundant is 30.

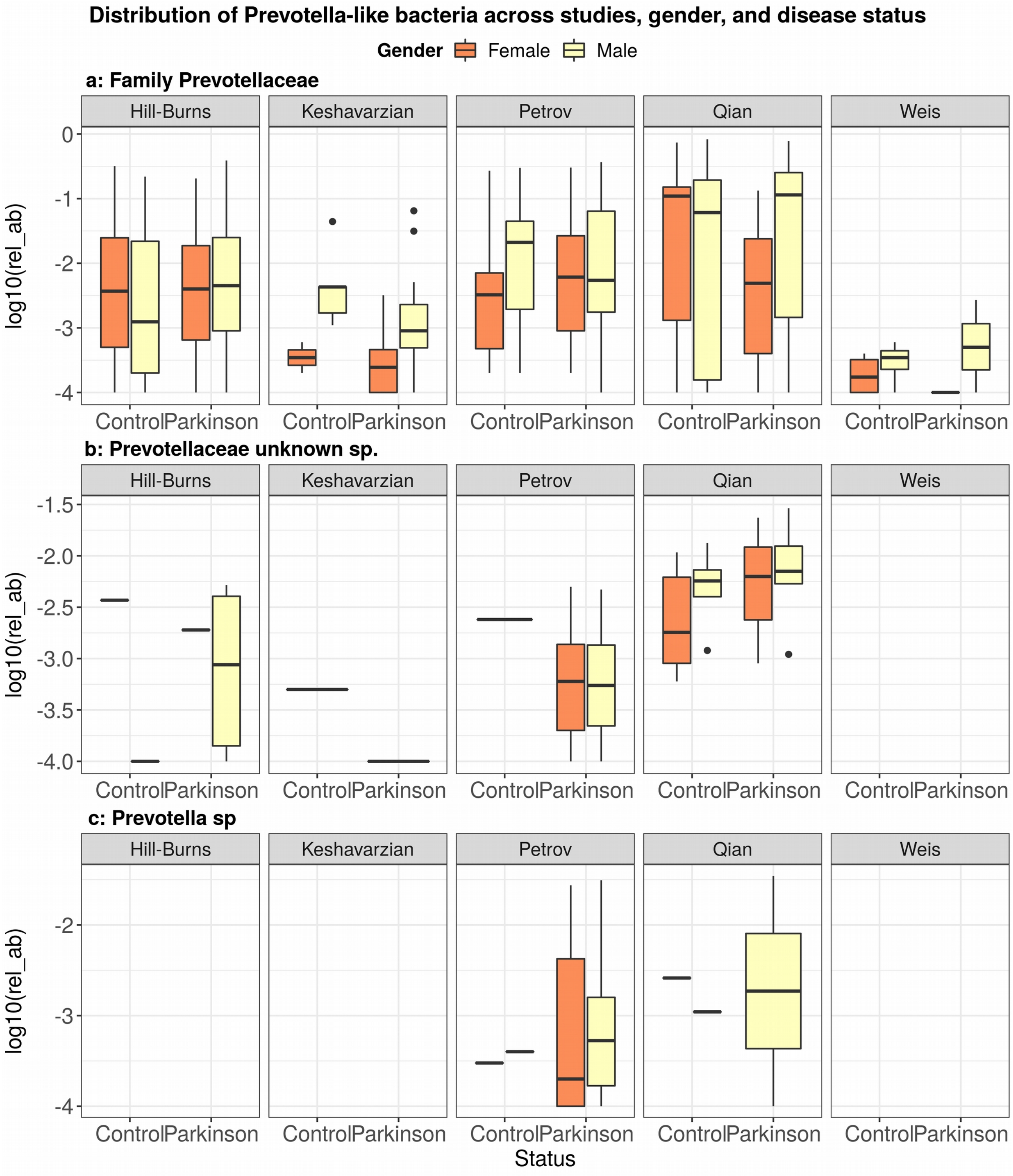

Supplementary figure 12 | Distribution of the family Prevotellaceae and species within this family across studies, disease status, and gender. Only data for the five studies reporting metadata are shown. The abundances of these taxa vary between genders across the sampling cohorts. This was observed for the Prevotellaceae family (**a**) and two species within this family (**b**, **c**).

Sensitivity analysis performed omitting the dataset from Scheperjans et al.

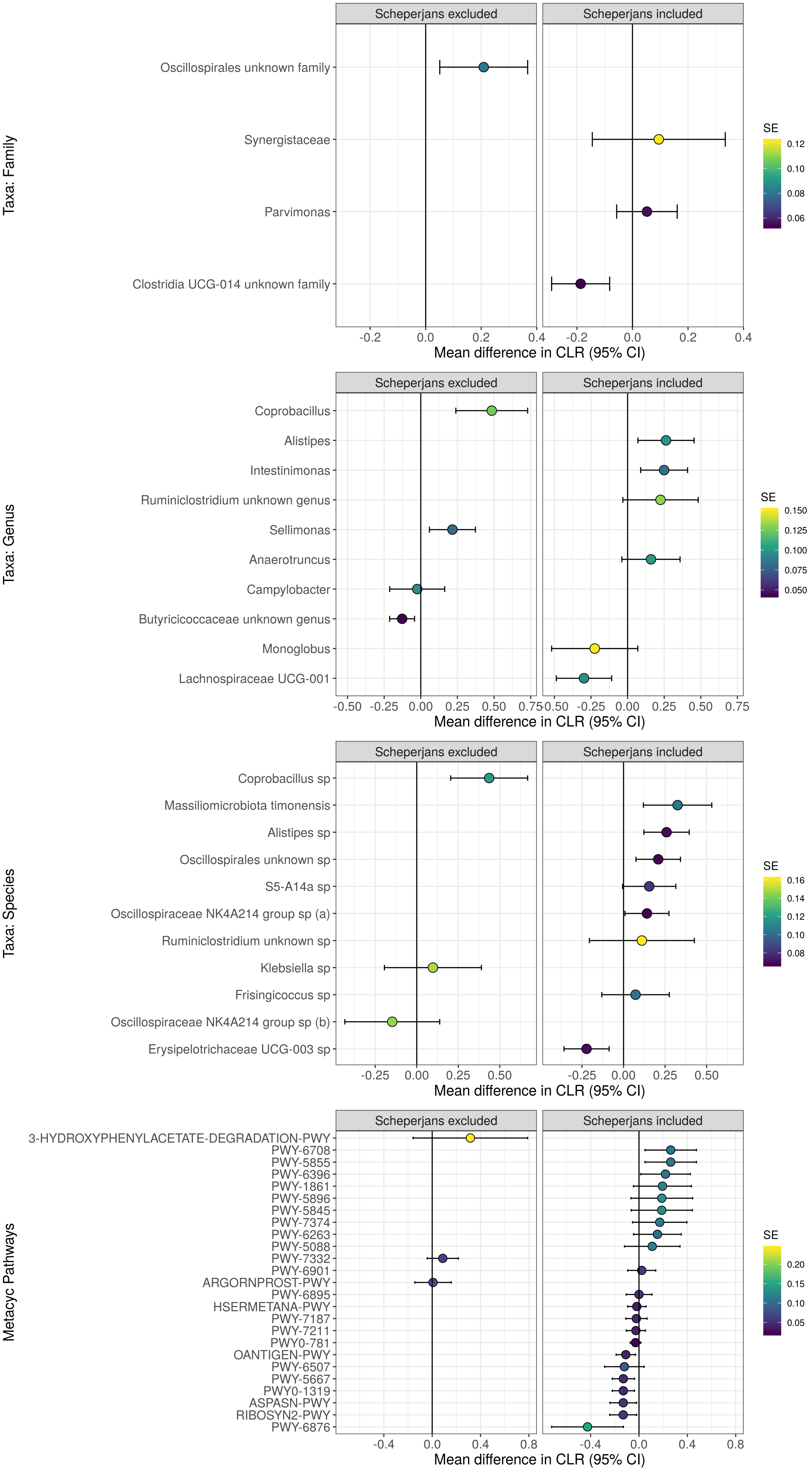

Supplementary Figure 13 | Results of the sensitivity analysis performed omitting the dataset of Scheperjans et al. The datasets of Scheperjans et al. and Aho et al. represent the baseline and follow-up of a longitudinal study performed in Finland. The differential abundance analyses we performed were repeated omitting the baseline dataset and the differences are reported here. Effect size and confidence intervals were calculated using the Pooled results approach. The color of the dots indicates the standard error of the estimate. Taxa and pathways more abundant in controls have an effect size shifted to the left, whereas taxa and pathways more abundant in PD have an effect size shifted to the right. Only taxa and pathways showing an overall small effect size were influenced by the presence of the baseline samples of the Finnish cohort.

Pathways showing significant differences in abundance between PD and controls

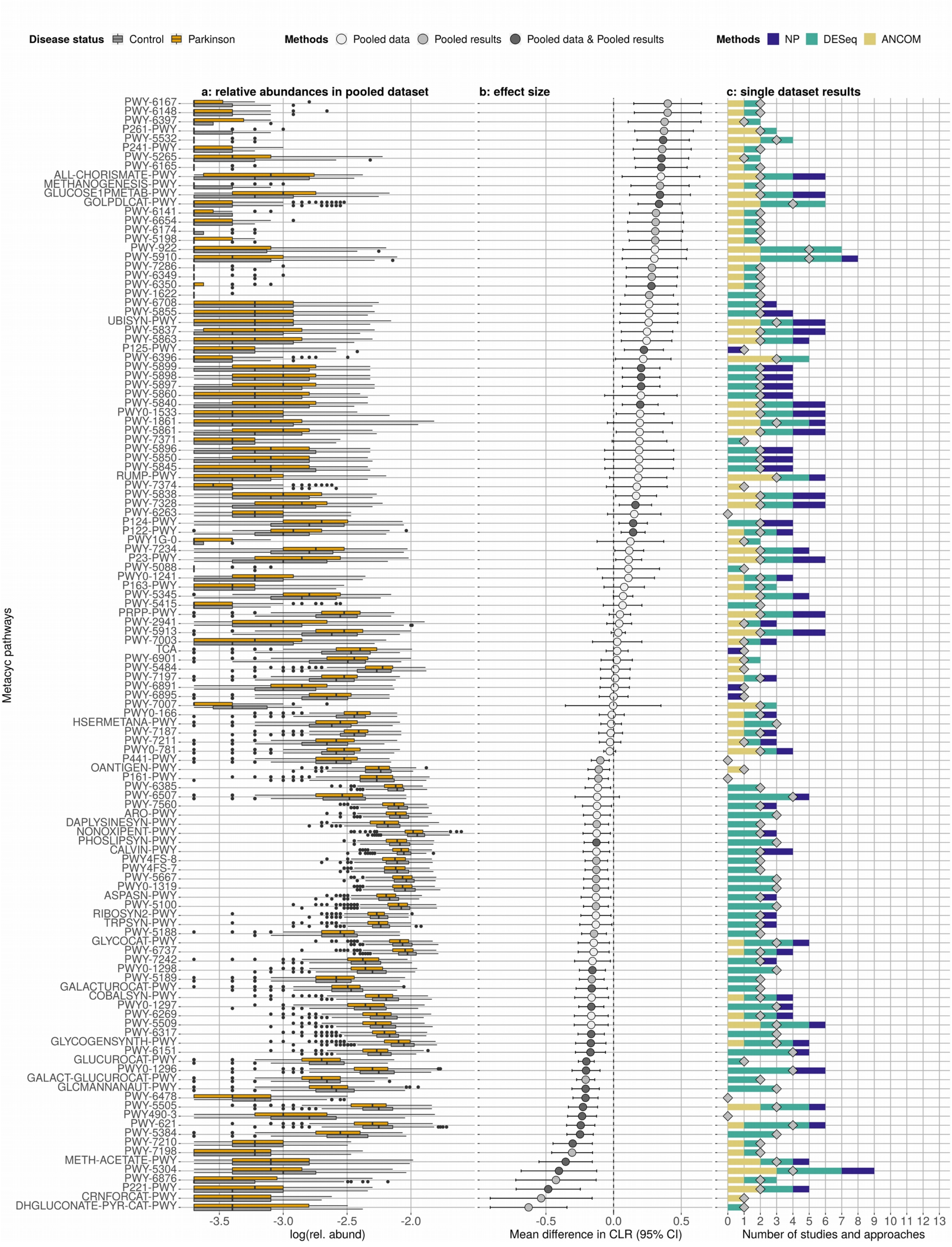

Supplementary Figure 14 | MetaCyc pathways showing a significant difference in abundance between PD patients and controls. The relative abundances of the pathways retrieved from the rarefied pooled data are reported in panel **a**. Effect sizes were estimated via the mean difference in CLR (panel **b**) using a random effect meta-analysis approach (Pooled results approach). This was calculated for all pathways resulting differentially abundant in the Pooled results or Pooled data approaches. The color of the dots indicates which of the above two approaches detected the pathway differentially abundant. Pathways more abundant in controls have an effect size shifted to the left, whereas pathways more abundant in PD have an effect size shifted to the right. Panel **c** shows the number of times each pathway was detected differentially abundant between PD patients and controls across studies (diamonds) and approaches (bars). We used ten studies and three approaches, hence the maximum number of times a pathway can be detected differentially abundant is 30.

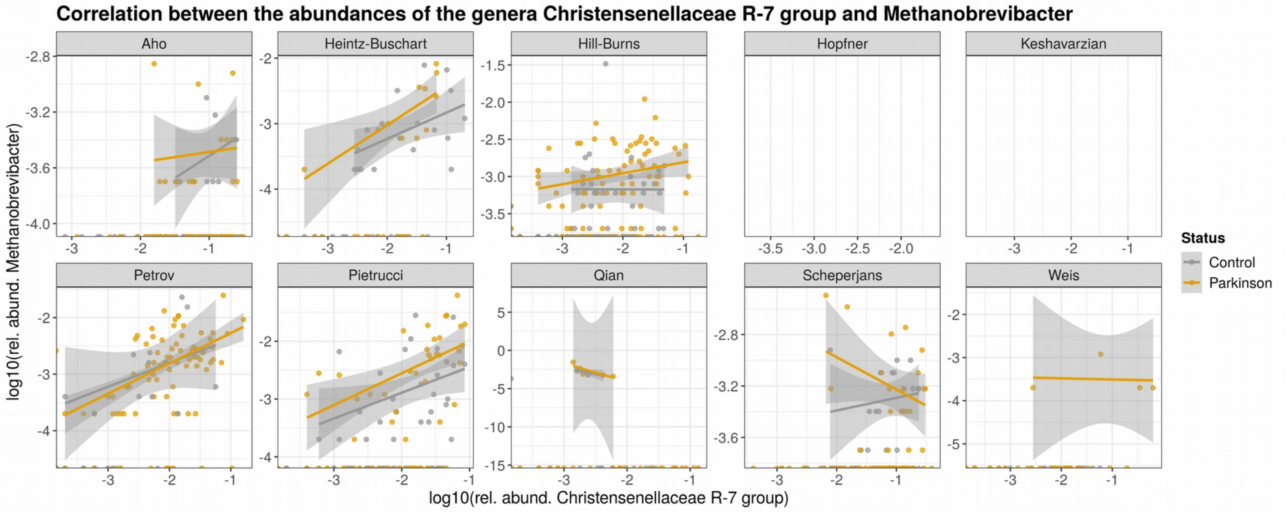

Supplementary Figure 15 | Correlation between the abundances of the genera Christensenellaceae R-7 group and *Methanobrevibacter* across studies. Data refer to abundances calculated on non-rarefied data. The genus *Methanobrevibacter* was not detected in the datasets of Hopfner et al. and Keshavarzian et al.

Supplementary table

Article	Method	Included in meta-analysis?	Comment
Heintz-Buschart et al.[1]	16S amplicons	Yes	
Hill-Burns et al.[2]	16S amplicons	Yes	
Petrov et al.[3]	16S amplicons	Yes	
Aiqun Lin et al.[4]	16S amplicons	No	
Yiwei Qian et al.[5]	16S amplicons	Yes	
Wei Li et al.[6]	16S amplicons	No	
Barichella et al.[7]	16S amplicons	No	
Keshavarzian et al.[8]	16S amplicons	Yes	
Pietrucci et al.[9]	16S amplicons	Yes	
Chin-Hsien Lin et al.[10]	16S amplicons	No	
Chunxiao Li et al.[11]	16S amplicons	No	
Aho et al.[12]	16S amplicons	Yes	
Fang Li et al.[13]	16S amplicons	No	
Baldini et al.[14]	16S amplicons	No	Pre-print only
Weis et al.[15]	16S amplicons	Yes	
Miao Jin et al.[16]	16S amplicons	No	
Heinzel et al.[17]	16S amplicons	No	Pre-print only
Gorecki et al.[18]	16S amplicons	No	
Hegelmainer et al.[19]	16S amplicons	No	
Wallen et al.[20]	16S amplicons	No	Pre-print only
Hopfner et al.[21]	16S amplicons	Yes	
Scheperjans et al.[22]	16S amplicons	Yes	These samples were re-sequenced by Aho et al.
Bedarf et al.[23]	Shot-gun metagenomics	No	

Supplementary Table 1 | List of studies that analyzed the gut microbiome in PD using amplicon sequencing or metagenomic approaches. The list results from a bibliography search performed in March 2020.

References

1. Heintz-Buschart, A. *et al.* The nasal and gut microbiome in Parkinson's disease and idiopathic rapid eye movement sleep behavior disorder: Nose and Gut Microbiome in PD and iRBD. *Mov. Disord.* **33**, 88–98 (2018).
2. Hill-Burns, E. M. *et al.* Parkinson's disease and Parkinson's disease medications have distinct signatures of the gut microbiome: PD, Medications, and Gut Microbiome. *Mov. Disord.* **32**, 739–749 (2017).
3. Petrov, V. A. *et al.* Analysis of Gut Microbiota in Patients with Parkinson's Disease. *Bull. Exp. Biol. Med.* **162**, 734–737 (2017).
4. Lin, A. *et al.* Gut microbiota in patients with Parkinson's disease in southern China. *Parkinsonism Relat. Disord.* **53**, 82–88 (2018).

5. Qian, Y. *et al.* Alteration of the fecal microbiota in Chinese patients with Parkinson’s disease. *Brain. Behav. Immun.* **70**, 194–202 (2018).
6. Li, W. *et al.* Structural changes of gut microbiota in Parkinson’s disease and its correlation with clinical features. *Sci. China Life Sci.* **60**, 1223–1233 (2017).
7. Barichella, M. *et al.* Unraveling gut microbiota in Parkinson’s disease and atypical parkinsonism. *Mov. Disord.* **34**, 396–405 (2019).
8. Keshavarzian, A. *et al.* Colonic bacterial composition in Parkinson’s disease: COLONIC MICROBIOTA IN PARKINSON’S DISEASE. *Mov. Disord.* **30**, 1351–1360 (2015).
9. Pietrucci, D. *et al.* Dysbiosis of gut microbiota in a selected population of Parkinson’s patients. *Parkinsonism Relat. Disord.* **65**, 124–130 (2019).
10. Lin, C.-H. *et al.* Altered gut microbiota and inflammatory cytokine responses in patients with Parkinson’s disease. *J. Neuroinflammation* **16**, 129 (2019).
11. Li, C. *et al.* Gut Microbiota Differs Between Parkinson’s Disease Patients and Healthy Controls in Northeast China. *Front. Mol. Neurosci.* **12**, (2019).
12. Aho, V. T. E. *et al.* Gut microbiota in Parkinson’s disease: Temporal stability and relations to disease progression. *EBioMedicine* **44**, 691–707 (2019).
13. Li, F. *et al.* Alteration of the fecal microbiota in North-Eastern Han Chinese population with sporadic Parkinson’s disease. *Neurosci. Lett.* **707**, 134297 (2019).
14. Baldini, F. *et al. Parkinson’s disease-associated alterations of the gut microbiome can invoke disease-relevant metabolic changes*. http://biorxiv.org/lookup/doi/10.1101/691030 (2019) doi:10.1101/691030.
15. Weis, S. *et al.* Effect of Parkinson’s disease and related medications on the composition of the fecal bacterial microbiota. *Npj Park. Dis.* **5**, 28 (2019).
16. Jin, M. *et al.* Analysis of the Gut Microflora in Patients With Parkinson’s Disease. *Front. Neurosci.* **13**, 1184 (2019).
17. Heinzel, S. *et al. Gut microbiome signatures of risk and prodromal markers of Parkinson’s disease*. http://biorxiv.org/lookup/doi/10.1101/2019.12.11.872481 (2019) doi:10.1101/2019.12.11.872481.
18. Gorecki, A. M. *et al.* Altered Gut Microbiome in Parkinson’s Disease and the Influence of Lipopolysaccharide in a Human α-Synuclein Over-Expressing Mouse Model. *Front. Neurosci.* **13**, 839 (2019).
19. Hegelmaier, T. *et al.* Interventional Influence of the Intestinal Microbiome Through Dietary Intervention and Bowel Cleansing Might Improve Motor Symptoms in Parkinson’s Disease. *Cells* **9**, 376 (2020).
20. Wallen, Z. D. *et al.* Characterizing dysbiosis of gut microbiome in PD: Evidence for overabundance of opportunistic pathogens. *bioRxiv* 2020.01.13.905166 (2020) doi:10.1101/2020.01.13.905166.
21. Hopfner, F. *et al.* Gut microbiota in Parkinson disease in a northern German cohort. *Brain Res.* **1667**, 41–45 (2017).
22. Scheperjans, F. *et al.* Gut microbiota are related to Parkinson’s disease and clinical phenotype. *Mov. Disord.* **30**, 350–358 (2015).
23. Bedarf, J. R. *et al.* Functional implications of microbial and viral gut metagenome changes in early stage L-DOPA-naïve Parkinson’s disease patients. *Genome Med.* **9**, (2017).

Supplementary data legends

Supplementary Data 1 | Proportion (R^2) of the gut microbiome data variability explained by study-specific factors and disease status. Estimates were obtained from dbRDAs performed constraining the data for each factor listed. The statistical significance of the constrain was tested using an ANOVA-like permutation test.

Supplementary Data 2 | Taxa and pathways having a significant difference in abundance between PD patients and controls in the individual datasets. Three independent statistical approaches were used: non parametric Wilcoxon-Mann-Whitney test on TSS normalized data, DESeq2, and ANCOM. The DESeq2 approach calculates the overall mean of the taxa abundance (baseMean), the fold change between conditions and the relative standard error (log2FoldChange and lfcSE), and performs statistical testing using BH p-value corrections. The W statistic and the significance for the different percentiles are reported for the ANCOM.

Supplementary Data 3 | Differentially abundant taxa between PD patients and controls as inferred by the Pooled data and Pooled results approaches. In the Pooled data approach we used the three methods applied to the individual datasets and selected only the taxa detected differentially abundant in two out of three methods. The DESeq2 approach calculates the overall mean of the taxa abundance (baseMean), the fold change between conditions and the relative standard error (log2FoldChange and lfcSE), and performs statistical testing using BH p-value corrections. The W statistic and the significance for the different percentiles are reported for the ANCOM.

Supplementary Data 4 | Results of the generalized linear mixed model (GLMM) comparisons performed to assess the effect of age and/or gender on taxa abundances. GLMMs using various factor interactions were created and their quality was assessed using AIC. If amongst the models with a ΔAIC<2, a model not containing the disease status was present we considered the disease status as not essential to explain the abundance of the taxon. Otherwise, we concluded that the disease status was an essential factor shaping taxa abundances. The model names refer to: full, ~ _status*age + status*gender + (1 + status|study)_; no int, ~ _status + gender + age + (1 + status|study)_; no int no random, ~ _status + gender + age + (1|study)_; no status, ~ _gender + age + (1|study)_; only status, ~ _status + (1 + status|study)_; only status no random, ~ _status + (1|study)_; no age, ~ _status*gender + (1 + status|study)_; no age no int, ~ _status + gender + (1 + status|study)_; no age no random, ~ _status*gender + (1|study)_; no age no random no int; ~ _status + gender + (1|_

study); no age int, ~ *status*gender + age + (1 + status|study)*; no age int no random, ~ *status*gender + age + (1|study);* only age, ~ *age + (1|study)* . To assess the effect of gender we then used the same model schema reported above for age.

Supplementary Data 5 | Parameters used to run the *sdm* program called by Lotus.
